# The Molecular Mechanisms of Regulating Oxidative Stress-Induced Ferroptosis and Therapeutic Strategy in Tumors

**DOI:** 10.1155/2020/8810785

**Published:** 2020-12-21

**Authors:** Jinghan Zhu, Yixiao Xiong, Yuxin Zhang, Jingyuan Wen, Ning Cai, Kun Cheng, Huifang Liang, Wanguang Zhang

**Affiliations:** ^1^Hepatic Surgery Center, Tongji Hospital, Tongji Medical College, Huazhong University of Science and Technology, Wuhan, Hubei 430030, China; ^2^Hubei Key Laboratory of Hepato-Pancreato-Biliary Diseases, Wuhan, Hubei 430030, China; ^3^Key Laboratory of Organ Transplantation, Ministry of Education, NHC Key Laboratory of Organ Transplantation, Key Laboratory of Organ Transplantation, Chinese Academy of Medical Sciences, Wuhan, Hubei 430030, China

## Abstract

Ferroptosis is an atypical form of regulated cell death, which is different from apoptosis, necrosis, pyroptosis, and autophagy. Ferroptosis is characterized by iron-dependent oxidative destruction of cellular membranes following the antioxidant system's failure. The sensitivity of ferroptosis is tightly regulated by a series of biological processes, the metabolism of iron, amino acids, and polyunsaturated fatty acids, and the interaction of glutathione (GSH), NADPH, coenzyme Q10 (CoQ10), and phospholipids. Elevated oxidative stress (ROS) level is a hallmark of cancer, and ferroptosis serves as a link between nutrition metabolism and redox biology. Targeting ferroptosis may be an effective and selective way for cancer therapy. The underlying molecular mechanism of ferroptosis occurrence is still not enough. This review will briefly summarize the process of ferroptosis and introduce critical molecules in the ferroptotic cascade. Furthermore, we reviewed the occurrence and regulation of reduction-oxidation (redox) for ferroptosis in cancer metabolism. The role of the tumor suppressor and the epigenetic regulator in tumor cell ferroptosis will also be described. Finally, old drugs that can be repurposed to induce ferroptosis will be characterized, aiming for drug repurposing and novel drug combinations for cancer therapy more efficiently and economically.

## 1. Introduction

Ferroptosis is an atypical form of programed cell death first proposed by Dixon et al. in 2012 [1] and is different from apoptosis, pyroptosis, autophagy, or the other types of cell death in morphology, biochemistry, and genetics. Morphologically, it is characterized by diminished mitochondrial cristae, ruptured mitochondrial outer membrane, and the shrunken mitochondria [1, 2]. The significant markers of ferroptosis in biochemistry are iron dependence and accumulation of lipid reactive oxygen species (ROS). An emerging evidence shows that oncogenes and tumor suppressors' change both have essential impacts on tumor cell ferroptosis regulation. For example, p53 has a dual role as pro- and antiferroptotic functions in response to oxidative stress [3, 4]. The tumor suppressor BRCA1-associated protein 1 (BAP1) represses SLC7A11 expression via reducing H2Aub occupancy on SLC7A11 promoter in a deubiquitinating-dependent manner [5]. The retinoblastoma (Rb) protein's status can modulate the responsiveness of HCC cells to sorafenib [6]. Clear cell carcinomas (CCCs) are vulnerable to ferroptosis due to its unique metabolic state [7]. The current conventional view is that the induction of ferroptosis is related to the regulation of system Xc^−^, iron sequestration, GSH metabolism, the activity of glutathione peroxidase 4 (GPX4), and finally imbalanced ROS homeostasis. A recent evidence shows that ferroptosis suppressor protein 1 (FSP1) repressed ferroptosis independent to the classical glutathione-based GPX4 pathway, in which through nonmitochondrial CoQ10 antioxidant system [8, 9]. Accordingly, ferroptosis is a nexus closely linked to metabolism, redox biology, and cancers. Cancer cells are characterized by increased aerobic glycolysis (Warburg effect) and elevated oxidative stress [10]. The regulation of antioxidant homeostasis is essential in maintaining cancer cell survival and normal cellular function. The occurrence of oxidative stress-induced ferroptosis is the consequence of imbalanced redox homeostasis. Although cancer cell survives in a high oxidative stress context, ferroptosis does not happen very often because of elevated antioxidant defense mechanisms of cancer cells [11].

For many years, it was generally considered that tumor initiation and progression depend on the oncogene activation or the tumor suppressor gene's inactivation. However, an emerging evidence shows that the ability to adapt to aberrant metabolism and escape from immunosurveillance is also essential for tumor cell survival [12]. Thus, targeting the “cart” (metabolism, immune) rather than the “horse” (oncogenes and tumor suppressors) may provide a new horizon for cancer therapy efficiently and selectively.

In the context of tumor microenvironments, tumor cells can overgrow because they evolved to be insensitive to the oxidative stress or the other harmful factors. As a consequence, tumor cells become resistant to stress inducers, such as chemotherapy and radiation. Here, ferroptosis can be a powerful tool to develop a new strategy by amplifying oxidative stress or inhibiting antioxidant regulators in tumor cells.

In this review, we summarized the basic regulation in oxidative-induced ferroptosis and its latest advances firstly, and then, the old drugs which can be repurposed for proferroptotic anticancer therapy has been described, which is aimed at expanding the new indications for existing drugs and alleviating the shortage of medicine for drug-resistant patients [13].

## 2. Metabolic Reprogramming and Ferroptosis in Tumor Cells

To fulfill tumor cells' needs for continuous proliferation, tumor cell metabolism is elaborately reprogrammed, thus forming a complex but precise metabolic-redox circuit [14]. Tumor cell metabolism and redox signal can be regulated mutually, and tumor metabolic reprogramming leads to ROS accumulation and cellular oxidative stress [15]. The main sources of ROS which can be used to trigger lipid peroxidation and ferroptosis include NADPH oxidase- (NOX-) induced ROS and mitochondrial-derived ROS (mROS) [16]. To relieve oxidative stress, tumor cells can also control the intracellular antioxidant system (GSH, NADPH, et al.).

The intracellular accumulation of ROS is a double-edged sword for tumor progression [17]. The redox status of cancer cells usually differs from that of normal cells. ROS are generally considered toxic substances in the cell. Because of metabolic and genetic aberration, cancer cells exhibit upregulated ROS levels. Adapting to stress helps tumor cells evolved powerful ROS scavenging systems to maintain redox homeostasis. This dynamic equilibrium makes ROS as a promoter in tumor development and progression [17]. Therefore, the critical point of using ROS as a tool for anticancer therapy is to trigger it into a lethal level. This can be achieved by treating cancer cells either with ROS-inducing therapies or with antioxidant-inhibiting therapies, which are new strategies that can kill cancer cells effectively and selectively.

Understanding the specific pathways involved in ROS and ferroptosis is essential to develop therapeutic approaches for cancer therapy. Below, we have summarized the significant modulators of antioxidant gene expression and ferroptotic pathway they mediate.

## 3. Regulation of Oxidative Stress-Induced Tumor Cell Ferroptosis

**Figure 1:**
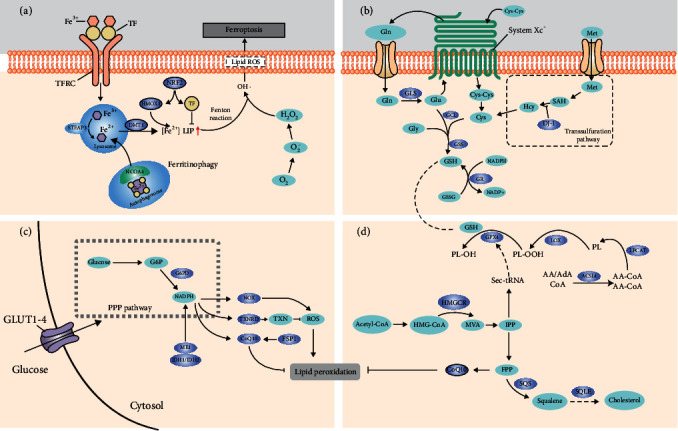
The central metabolic regulation of ferroptosis. (a) Transferrin, TFRC, and NCOA4 regulate iron sequestration by increasing the levels of [Fe^2+^] LIP, thus triggering lipid peroxidation and ferroptosis. (b) System Xc^−^ (composed by SLC7A11 and SLC3A2) exchanges intracellular glutamate for extracellular cystine, thereby supporting intracellular glutathione (GSH) synthesis, which is the primary endogenous antioxidant. GPX4 uses GSH to detoxify lipid hydroperoxide, thus protecting tumor cells against ferroptosis. And the transsulfuration pathway affects ferroptosis sensitivity by providing cysteine for GSH synthesis. (c) NADPH can be used for the regeneration of the reduced form of glutathione (GSH) by glutathione reductase (GR). There are three significant ways for intracellular NADPH production—produced from pentose phosphate pathway, produced by isocitrate dehydrogenase 1 (IDH1) and IDH2 through the conversion of isocitrate to *α*-ketoglutarate, and malic enzyme 1 (ME1) through the transformation of malate to pyruvate. In addition to GSH regeneration, the regeneration of thioredoxin (TXN) and the activity of CoQ10 are also required for the participation of NADPH to eliminate ROS and antiferroptosis. And in some cellular contexts, NADPH can be oxidized by NADPH oxidase (NOX), thus increasing intracellular ROS levels. (d) Isopentenyl pyrophosphate (IPP) produced from the mevalonate pathway is required for the cholesterol biosynthesis, Sec-tRNA, and CoQ10 production, which all can be used to regulate ferroptosis sensitivity. Sec-tRNA is essential for the integration of GPX4 with selenocysteine. TF: transferrin; DMT1: divalent metal transporter 1; TFRC: transferrin receptor; STEAP1: STEAP family member 1; NCOA4: nuclear receptor coactivator 4; LIP: labile iron pool; GLS: glutaminase; GCL: glutamate-cysteine ligase; GSS: glutathione synthetase; GR: glutathione reductase; DJ-1: also known as Parkinsonism-associated deglycase: PARK7; G6PD: glucose-6-phosphate dehydrogenase; ME1: malic enzyme 1; IDH1/IDH2: isocitrate dehydrogenase 1/2; NOX: NADPH oxidase; TXNRD: thioredoxin reductase 1; CoQ10: coenzyme Q10; TXN: thioredoxin; HMGCR: 3-hydroxy-3-methylglutaryl-CoA reductase; MVA: mevalonate; IPP: isopentenyl pyrophosphate; FPP: farnesyl pyrophosphate; SQS: squalene synthase; SQLE: squalene monooxygenase; GPX4: glutathione peroxidase 4; LOX: lipoxygenase; ACSL4: acyl-CoA synthetase long-chain family member 4; LPCAT3: lysophosphatidylcholine acyltransferase 3.

It is currently considered that ferroptosis is related to iron, amino acids, and lipid metabolism, but the sensitivity of ferroptosis is also modulated by several noncanonical pathways (Figure 1). In the following, we first summarized the role of iron sequestration in ferroptosis, which can form ROS, leading to cell damage. Next, we describe the GSH metabolism and NADPH metabolism, which act as ROS scavenger and reduce oxidized phospholipids (PLs). Last, we described the role of lipid metabolism that provides substrates for lipid peroxidation and the other ferroptotic effector's function, including transcription factors, mevalonate pathway, and epigenetic regulator in ferroptosis.

### 3.1. Regulation of Iron Sequestration in Oxidative-Induced Ferroptosis

Iron is a redox-active metal that is facilitated for the ROS production by Fenton reaction. Numerous studies show that genes involved in iron homeostasis impact ferroptosis sensitivity (Figure 1(a)). Inactivation of NF2, a tumor suppressor, rendered cancer cells more sensitive to ferroptosis by NF2-YAP axis through upregulating the expression of transferrin receptor (TFRC) and acyl-CoA synthetase long-chain family member 4 (ACSL4) [18], while genetic inhibition of the iron metabolism key regulator (IREB2) causes tumor cells resistant to ferroptosis [1]. Repression of nitrogen fixation 1 (NFS1) predisposes tumor cells to ferroptosis by activating the iron-starvation response via stabilizing TFRC transcription and inhibiting ferritin heavy chain 1 (FTH1) translation [19]. The degradation of FTH1 in the lysosome can increase the accumulation of iron in the cell. Genetic inhibition of NCOA4 (nuclear receptor coactivator 4), a cargo receptor that recruits FTH to autophagosome from lysosome [20], abrogates ferritin degradation and ROS accumulation, thus inhibiting ferroptosis occurrence. Except for the direct effect of nuclear factor erythroid 2-related factor 2 (NRF2) as a ROS scavenger via GSH metabolism, it can also regulate ROS levels by modulating free Fe^2+^ homeostasis [21]. The main source of intracellular free Fe^2+^ is derived from the breakdown of ferritin by lysosome or the degradation of haem by haem oxygenase (HMOX1); nuclear factor erythroid 2-related factor 2 (NRF2) positively regulates HMOX1 transcription [22]. This seems a bit paradoxical, as a well-recognized ferroptosis suppressor NRF2 would promote uncontrolled Fe into cells that facilitate Fenton reaction. Interestingly, except for HMOX1 elevation, NRF2 enhanced the transcription of ferritin light chain (FTL) and FTH, two components of the ferritin complex, which can detoxify free Fe^2+^ and soon store it in its own structure [23, 24]. Accordingly, NRF2 reduces ROS production by hindering Fe(II) released from haem and its subsequent chelation. The sustained activation of NRF2 leads to iron signaling alteration, which enhances tumor cell's resistance to ROS and is beneficial to tumor cell survival and progression.

### 3.2. Redox Regulation of GSH Metabolism

#### 3.2.1. Regulation of Ferroptosis by GPX4

GSH metabolism and NADPH synthesis's capacity is commonly considered as positively correlating with ferroptosis sensitivity (Figures 1(b) and 1(c)). Glutathione peroxidase 4 (GPX4) is the only enzyme that can reduce phospholipid peroxides (oxidized phosphatidylethanolamine (PE) and phosphatidylcholine (PC)) directly [25]. GSH was found as a cofactor of GPX4 [26]; GPX4 uses two molecules of GSH to reduce phospholipid peroxides; the enzyme activity of GPX4 is directly correlated to GSH metabolism. So, that is why GPX4 and GSH play an essential role in ferroptosis regulation. Pharmacological inhibition by (1S,3R)-RSL3 or genetical GPX4 silencing can induce ferroptosis in vitro and in vivo [2], and the adaptor protein 14-3-3*ε* is required for the RSL3-induced ferroptosis [27]. Zou et al. reported that clear cell carcinomas (CCCs) are vulnerable to ferroptosis because of its unique metabolic state, in which hypoxia-inducible factor 2*α* selectively enriches polyunsaturated lipids, by HILPDA (hypoxia-inducible, lipid droplet-associated protein) activation [7]. Data analysis shows that GPX4 is highly expressed in tumor tissues, compared with adjacent tissues. The upstream of GPX4 is characterized by lower DNA methylation sites and elevated H3K4me3 and H3K27ac levels, suggesting that enhanced expression of GPX4 in tumor tissues may result from epigenetic regulation [28].

### 3.3. Regulation of Ferroptosis by System Xc^−^

**Figure 2:**
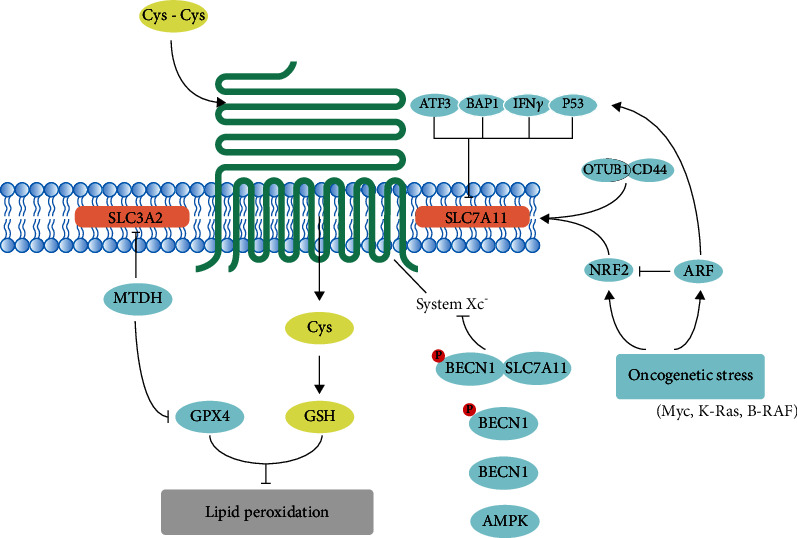
Modulation of system Xc^−^ in ferroptotic cancer cells.The cystine/glutamate exchanger is composed of SLC7A11 and SLC3A2. Inhibition of them both can impair the uptake of cystine and following GSH synthesis, thus affect the activity of GPX4 and sensitize tumor cells to ferroptosis. Cys-Cys: cystine; Cys: cysteine; GSH: glutathione; SLC3A2: solute carrier family 3 member 2; SLC7A11: solute carrier family 7 member 11; MTDH: metadherin; ATF3: activating transcription factor 3; BAP1: BRCA1-associated protein 1; IFN*γ*: interferon-*γ*; p53: tumor protein p53; OTUB1: OTU deubiquitinase, ubiquitin aldehyde binding 1; CD44: CD44 molecule (Indian blood group); NRF2: nuclear factor erythroid 2-related factor 2; ARF: also known as CDKN2A: cyclin-dependent kinase inhibitor 2A; BECN1: beclin1; AMPK: also known as PRKAA2: protein kinase AMP-activated catalytic subunit alpha 2.

Reduced glutathione (GSH) is an essential antioxidant within cells that can prevent ferroptosis by attenuating ROS accumulations. The levels of extracellular cystine and intracellular cysteine are required for the GSH synthesis, thus correlated to the enzyme activity of GPX4 and the sensitivity to ferroptosis. The cystine-glutamate antiporter, system Xc^−^, is composed by two subunits, SLC3A2 and SLC7A11. Inhibition of system Xc^−^ impairs the intake of cystine thus decreasing GSH synthesis and following lipid peroxidation and ferroptosis (Figure 2).

SLC7A11 is the catalytic subunit of system Xc^−^ and highly specific to cystine and glutamate, and the regulatory subunit SLC3A2 primarily functions as a chaperone protein that is essential to regulate retention of SLC7A11 on plasma membrane [1, 29]. It has been reported that SLC3A2 deletion results in a downregulation of SLC7A11 levels, indicating that SLC3A2 is required to maintain SLC7A11 stability and its deficiency will impair the integrity of the system Xc^−^, thus sensitizing tumor cells to ferroptosis [30]. The efficient exchange of cystine and glutamate by system Xc^−^ requires both catalytic subunit and regulatory subunit.

Emerging evidences show that many factors can induce ferroptosis by system Xc^−^ inhibition. As a typical stress sensor, activating transcription factor 3 (ATF3) can promote erastin-induced ferroptosis via binding to the promoter region of SLC7A11 and suppressing SLC7A11 expression in a p53-independent manner [31]. SLC7A11 is an essential BAP1 target in human cancers, and BAP1 represses SLC7A11 expression via reducing H2Aub occupancy on SLC7A11 promoter in a deubiquitinating-dependent manner [5, 32]. After phosphorylated by isoflurane or AMPK activation, Beclin1 (BECN1) can bind to SLC7A11, blocking system Xc^−^ activity [33, 34]. Wang et al. reported that interferon-*γ* released from CD8^+^ T cells downregulates the expression of SLC3A2 and SLC7A11 and, as a consequence, promotes tumor cell lipid peroxidation and ferroptosis [35], suggesting that the selective killing ability of CD8^+^ T cells for tumor cells is partially due to ferroptosis induced by interferon-*γ*. NRF2 can transcriptionally activate SLC7A11, whereas ARF (also known as CDKN2A) negatively regulates NRF2's activity in a p53-independent manner, indicating that NRF2 is a major target of p53-independent tumor suppression by ARF [36]. Expression of suppressor of cytokine signaling 1 (SOCS1) sensitized cells to ferroptosis via a p53-dependent manner; expression of SOCS1 reduced the levels of GSH [37]. The role of p53 in ferroptosis by regulating SLC7A11 expression is presented in the following paragraphs. OTUB1 (OTU deubiquitinase, ubiquitin aldehyde binding 1) stabilizes SLC7A11 transcription through the deubiquitination of SLC7A11, which can be enhanced by CD44 [38]. KRAS mutation is a major oncogenic driver for lung adenocarcinoma (LUAD); mutationally activated KRAS strikingly increased SLC7A11 expression in an NRF2-dependent manner [39].

Hangauer et al. found that the survival of therapy-resistant high-mesenchymal cancers relied on the activity of GPX4 [40] and is especially vulnerable to ferroptosis inducers (sorafenib, erastin, and sulfasalazine); the underlying mechanism is that metadherin (MTDH) conferred to this therapy-resistant cell state and sensitizes cells to ferroptosis inducer by decreasing GPX4 and SLC3A2 expressions [41]. System Xc^−^ intakes extracellular cystine into cells to sustain intracellular cysteine pools and then together with glutamate and glycine to promote GSH synthesis catalyzed by two enzymes, glutamate-cysteine ligase (GCL) and glutathione synthetase (GSS). Knockdown of glutamate-cysteine ligase catalytic (GCLC) subunit sensitized tumor cells to ferroptosis [42]. Another source of cystine is the transsulfuration pathway. The inhibition of DJ-1 (also known as Parkinsonism-associated deglycase, PARK7) enhances tumor cells' sensitivity to ferroptosis inducer via disrupting the formation of the S-adenosyl homocysteine hydrolase (SAHase) and impairing its activity [43].

### 3.4. Regulation of NADPH Production and Utilization in Ferroptosis

Reduced NADPH, an essential metabolic substrate, is crucial to many biological processes (Figure 1(c)). For example, the generation of GSH and thioredoxin (TXN), which are essential in eliminating peroxides [44], both required for the participation of NADPH. NADPH depletion will lead to a decreased level of GSH and TXN, thus promoting lipid ROS accumulation in biological membranes [45]. The production of NADPH is mainly derived from three ways within cells: through the pentose phosphate pathway (PPP) by glucose-6-phosphate dehydrogenase (G6PD), as well as the conversion of pyruvate to malate by malic enzyme 1 (ME1) and the conversion of isocitrate to *α*-ketoglutarate (*α*-KG) by isocitrate dehydrogenase (IDH) isoforms. IDH1/2 mutation reduces the affinity for isocitrate and increases the affinity for NADPH and *α*-KG, which blocks the conversion of isocitrate to *α*-KG and facilitates the conversion of *α*-KG to oncometabolite 2-hydroxyglutarate (2-HG) [46]. Accordingly, because of decreased levels of NADPH, IDH1/2-mutant cells become sensitized to ferroptosis inducer [47]. Interestingly, the frequency of IDH1/2 mutation was relatively high in acute myeloid leukemia (AML) and glioma [48, 49]; this suggests that tumor cells with these kinds of mutations may be well response to proferroptotic therapy.

To scavenge hydrogen peroxide or lipid hydroperoxide, GSH is oxidized to glutathione disulfide (GSSG) by ROS and GPX4, through the activity of glutathione reductase (GR) and the reducing agent NADPH. This process uses NADPH as the electron receptor derived from the pentose phosphate pathway (PPP). The PPP generates NADPH, which is essential for sustaining the cellular levels of GSH and resistance to ferroptosis. However, it can also supply NADPH to NADPH oxidases (NOXs) and contribute to ROS production and ferroptosis in some cellular contexts. The broad-spectrum NOX inhibitor diphenyleneiodonium and the selective NOX1/4 isoform inhibitor GKT137831 significantly diminished erastin-stimulated ROS, lipid ROS, and cell death [50]. By using large-scale testing of small molecules in 60 human cell lines combined with transcriptome analysis, Shimada et al. identified intracellular NADPH abundance negatively correlates with sensitivity to ferroptosis inducers [51]. NOXs, LOXs, and mitochondrial transport complexes all can fuel ROS production, targeting that these ROS-producing enzymes may be a new way to kill tumor cells selectively.

### 3.5. Redox Regulation of Lipid Metabolism in Ferroptosis

**Figure 3:**
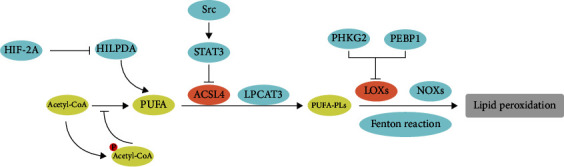
Modulation of lipid metabolism in ferroptotic cancer cells.Lipid metabolism is intimately involved in determining cell sensitivity to ferroptosis. PUFAs are susceptible to lipid peroxidation and are necessary for the execution of ferroptosis. ACSL4 and LPCAT3 are involved in the synthesis and remodeling of PUFA-PLs in cell membranes. Inhibition of ACSL4 and LPCAT3 depletes the substance for lipid peroxidation and increases resistance to ferroptosis. LOXs, NOXs, and Fenton reactions contribute to ROS production, thus promote ferroptosis. HIF-2A: hypoxia-inducible factor-2A; HILPDA: hypoxia-inducible, lipid droplet-associated protein; PUFA: polyunsaturated fatty acids; STAT3: signal transducer and activator of transcription 3; Src: SRC protooncogene, nonreceptor tyrosine kinase; PUFA-PLs: polyunsaturated fatty acids-phospholipids; PHKG2: phosphorylase kinase G2; PEBP1: phosphatidylethanolamine-binding protein 1.

Accumulation of phospholipid peroxides in biomembranes is the hallmark and rate-limiting step of ferroptosis (Figure 1(d) and Figure 3). Lipidomic analysis indicates that phosphatidylethanolamines (PEs) including arachidonic acid (AA) and its extended product, adrenic acid (AdA), are vital phospholipids that undergo oxidation and drive cells to ferroptosis [52, 53]. Acyl-CoA synthetase long-chain family member 4 (ACSL4) transforms AA or AdA to acylated AA or acylated AdA; lysophosphatidylcholine acyltransferase 3 (LPCAT3) catalyzes the acylated AA/AdA into PEs, while LOXs, NOXs, and Fenton reaction may oxidize AA-PE or AdA-PE to trigger ferroptosis [54]. Inhibition of these genes can remove the substrate for lipid peroxidation and make cells resistant to ferroptotic cell death [54, 55].

The integrin *α*6*β*4 can protect tumor cells from death in adverse conditions and is correlated to metastasis [56]. *α*6*β*4-mediated activation of Src (SRC protooncogene, nonreceptor tyrosine kinase) and STAT3 (signal transducer and activator of transcription 3) suppresses expression of ACSL4, thus inhibiting the occurrence of ferroptosis [57]. Deprivation of cystine led to several EGFR/MAPK-driven tumors more sensitized to ferroptosis, as GPX4 inhibition and catalase NOX4 at the same time [58]. Treatment of xenografts derived from EGFR-mutant non-small-cell lung cancer (NSCLC) with cysteinase (an engineered enzyme that can deplete both cysteine and cystine with cell) showed increased ferroptosis. Phosphorylase kinase G2 (PHKG2) can regulate iron availability of lipoxygenase, including arachidonate LOX12 (ALOX12), ALOX12B, ALOX15, and ALOX15B, which in turn promote ferroptosis through lipid peroxide accumulation. p53 activation modulates ferroptosis responses, while ALOX12 inactivation abrogated p53-dependent ferroptosis, indicating that ALOX12 is essential for p53-dependent tumor suppression [59]. Phosphatidylethanolamine-binding protein 1 (PEBP1), a scaffold protein inhibitor of protein kinase cascades, was shown to bind and direct LOX15 toward PUFAs in the cell membrane to promote ferroptosis [60]. Zou et al. reported cytochrome P450 oxidoreductase (POR) contributes to ferroptosis across a broad range of lineages and cell states. In response to distinct mechanisms of ferroptosis induction [61] and by using systematic lipidomic profiling, POR's activity was further mapped to the lipid peroxidation step in ferroptosis. Cancer cells with high-level AMP-activated protein kinase (AMPK) activation are resistant to ferroptosis. Further analyses show that AMPK regulation of ferroptosis is related to AMPK-mediated phosphorylation of acetyl-CoA carboxylase (ACC) and PUFA synthesis [62].

Oxidative stress-induced lipid peroxidation plays an essential role in ferroptosis. The fundamental mechanism is based on an overload of ROS, which attacks biomembranes, scatters lipid peroxidation chain reactions, and subsequently induces ferroptosis. Targeting molecules involved in the lipid peroxidation network can effectively and selectively induce ferroptosis, thus killing tumor cells.

### 3.6. The Other Regulators in Ferroptosis Modulation

#### 3.6.1. Role of Transcription Factors in Ferroptosis Regulation

BTB domain and CNC homolog 1 (BACH1), a transcription factor in heme and iron metabolism and a repressor of NRF2, promotes ferroptosis by suppressing the transcription of a cohort of proferroptotic genes. These sets of genes are involved in GSH biosynthesis or iron sequestration, which include FTH1, FTL1, SLC7A11, glutamate-cysteine ligase modifier subunit (GCLM), and solute carrier family 40 member 1 (SLC40A1) [63].

**Figure 4:**
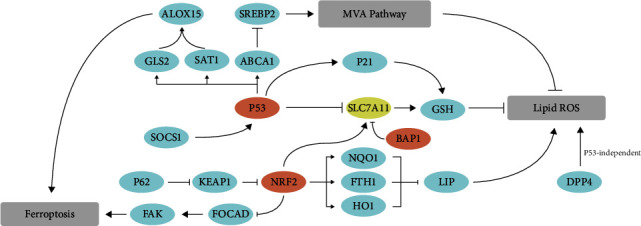
The role of transcription factors in ferroptosis. p53 has a dual function in the control of ferroptosis. On the one hand, p53 can promote ferroptosis via repression SLC7A11 expression or through promoting SAT1 and GLS2 expression, thus activating ALOX15 to induce lipid peroxidation and ferroptosis. On the other hand, the antiferroptotic function of p53 is that p53 can transcriptionally inhibit DPP4 expression or induction of p21 expression. NRF2 can negatively control ferroptosis sensitivity by activating SLC7A11 transcription, inhibiting cytosolic accumulation of labile iron pool, or repressing FOCAD expression, thus promoting tumor cells resistant to ferroptosis. ALOX15: arachidonate lipoxygenase 15; SREBP2: sterol regulatory element-binding transcription factor 2; GLS2: glutaminase 2; SAT1: spermidine/spermine N1-acetyltransferase 1; ABCA1: ATP-binding cassette subfamily A member 1; P21: also known as CDKN1A: cyclin-dependent kinase inhibitor 1A; SOCS1: suppressor of cytokine signaling 1; p63: tumor protein p63; KEAP1: Kelch-like ECH-associated protein 1; NQO1: NAD(P)H quinone dehydrogenase 1; FTH1: ferritin heavy chain 1; HO-1: also known as HMOX1: heme oxygenase 1; FOCAD: focadhesin; FAK: also known as PTK2: protein tyrosine kinase 2.

p53, encoded by the TP53 gene, plays a critical role in tumor suppression, and more than half of all cancers harbor p53 mutation or inactivation. Much evidence suggests that p53 controls multiple cellular processes at the transcription level, including cell cycle arrest, senescence, DNA repair, and apoptosis. However, the evidence is emerging that the function of p53 in the metabolic pathway is also essential. p53 has a controversial role in response to oxidative stress as it can promote both pro- and antiferroptotic responses (Figure 4 and Table 1) [3, 4].

Jiang et al. reported that an acetylation-defective mutant, p53^3KR^, in which three lysine residues were replaced by arginine (K117/161/162R), losses the ability to induce cell cycle arrest, senescence, and apoptosis, but still has the capability of generating ferroptosis via SLC7A11 inhibition, thus sensitizes osteosarcoma U2OS cells to ferroptosis [64]. Jiang et al. found that induction of ferroptosis was required for the tumor suppression function of p533KR in vitro and in vivo [64]. p53's functional N-terminal domain is essential for its capacity to regulate oxidative stress responses and ferroptosis [65]. Ou et al. reported that p53 could also regulate ferroptosis via p53-SAT1-ALOX15 axis [66]. Knockdown of SAT1 (spermidine/spermine N1-acetyltransferase 1) partially rescued ROS-induced ferroptosis. Other p53 variants such as p53^R273H^ and p53^R175H^, two commonly occurring p53 mutants, can also inhibit SLC7A11 expression by hampering NRF2-dependent SLC7A11 elevation [67–69]. Interestingly, as the oncogene MYC controls glutaminase 1 (GLS1) expression [70], the tumor suppressor p53 controls glutaminase 2 (GLS2) transcription, thus regulating ferroptosis via influencing GSH synthesis. [42]

Notably, p53 can negatively modulate ferroptosis in certain scenarios. The p53 stabilization can delay ferroptosis induction via sustaining glutathione in a p53-p21 (also known as CDKN1A)-dependent axis [71]. p53 blocks erastin-induced ferroptosis by inhibiting the expression of dipeptidyl peptidase 4 (DPP4). In p53-deficient cells, DPP4 formed a complex with NOX1 that mediates lipid peroxide accumulation and following ferroptosis [72].

NRF2 is generally considered the most significant modulator of intracellular antioxidant response [73]. Under unstressed conditions, NRF2 was sequestered by Kelch-like ECH-associated protein 1 (KEAP1) and then targeted degradation by the proteasome [74].

Upon exposure to oxidative stress, Keap1 can be modified by reactive cysteine residues, results in NRF2 detaches from KEAP1, and translocates into nucleus [75]. NRF2 controls the production of GSH by upregulating SLC7A11 expression. NRF2 silencing promotes hepatocellular carcinoma (HCC) cells sensitized to ferroptosis [76], suggesting that NRF2 plays a crucial role in protecting HCC against ferroptosis. Inhibition of Lon peptidase 1 (LONP1) mitochondrial protects pancreatic ductal adenocarcinoma cells against erastin-induced ferroptosis through inhibiting the KEAP1/NRF2 pathway and GPX4 downregulation [77]. NRF2 negatively regulates FAK activity by downregulating FOCAD expression, thus hampering ferroptosis in NSCLC cells. Erastin treatment combined with NRF2 inhibitor (brusatol) showed better therapeutic efficacy partially due to NRF2-FOCAD-FAK axis [78]. Yang et al. reported that sirtuin 2 (SIRT2) downregulates intracellular NRF2 levels via deacetylating NRF2 on lysine 506 and 508 [79]. The reduction of NRF2 decreased a series of iron metabolism-related gene expressions, including HMOX1, FTH, FTL, and ferroportin 1 (FPN1), which results in intracellular iron accumulation.

#### 3.6.2. Redox Regulation of the Mevalonate (MVA) Pathway

The MVA pathway is a well-accepted driving malignant transformation, and cancer cells deeply rely on some of the MVA pathway products [80]. The path is an enzymatic cascade responsible for de novo cholesterol synthesis (Figure 4). HMG-CoA reductase (HMGCR), the rate-limiting enzyme in the MVA pathway, catalyzes 3-hydroxy-3-methylglutaryl-coenzyme A (HMG-CoA) to MVA. The direct product of MVA, isopentenyl pyrophosphate (IPP), is essential for cholesterol biosynthesis, isopentenylation of Sec-tRNA, and CoQ10 production [81]. Inhibition of squalene synthase (SQS), an enzyme in the downstream of IPP, blocks ferroptosis. In contrast, FIN56, a specific inducer of ferroptosis, can bind to and activate SQS, thus promoting cholesterol synthesis and following increased sensitivity to ferroptosis [82]. Meanwhile, the inhibition of HMGCR by statins enhances ferroptosis sensitivity.

Despite cholesterol synthesis, the MVA pathway is also essential for GPX4 formation. There was selenocysteine on the GPX4 catalytic subunit, IPP, and Sec-tRNA are required for the insertion of Sec to GPX4 [83]. Selenium replenishment elevates ferroptosis resistance, while selenium depletion benefits ferroptosis sensitivity.

By activating the activity of SQS, FIN56 decreased the production of CoQ10, which in turn increased the sensitivity to ferroptosis. The underlying mechanism is that reduced CoQ10 acts as a lipophilic radical-trapping antioxidant (RTA) that halts the propagation of lipid peroxides [8, 9]. Bersuker et al. and Doll et al. found that ferroptosis suppressor protein 1 (FSP1) inhibits ferroptosis in a GPX4-independent pathway. The further study shows that FSP1 can act as an oxidoreductase to reduce CoQ10 by using NADPH, generating RTA that inhibits ferroptosis [8, 9]. In this connection, supplementation of cells with CoQ10 effectively suppresses ferroptosis [51]. MDM2 and MDMX, negative regulators of p53, also negatively regulated the levels of FSP1 in a p53-independent manner. This indicates that MDM2 and MDMX usually prevent cells from making enough defenses against lipid peroxidation and promoting ferroptosis [84].

#### 3.6.3. The Epigenetic Regulation of Redox-Induced Ferroptosis

Until now, there is a growing evidence that shows epigenetic regulation, including DNA methylation, histone modification, RNA methylation, and posttranslational modification (PTM), plays a significant role in cancer formation. Although some studies indicate the importance of epigenetic regulation in ferroptosis in recent years, the relationship between epigenetic mechanism and ferroptosis is still poorly defined. Here, we will list several significant epigenetic-related ferroptotic regulators including methylation and PTM.

GPX4 is highly expressed in tumor tissues, compared with adjacent tissues, and was inverse correlated with the patient's prognosis. Further analysis reveals that the upstream of GPX4 is characterized by lower DNA methylation sites and elevated H3K4me3 and H3K27ac levels, suggesting that a high level of GPX4 in cancer may result from epigenetic regulation [28]. Upregulated methylation of GPX4 inhibits its expression thus leading to lipid ROS accumulation and following ferroptosis [85].

Lymphoid-specific helicase (LSH), a member of the SNF2 family of chromatin remodeling ATPases encoded by the HELLS gene, is a critical DNA methylation modifier [86]. Jiang et al. reported that LSH inhibits ferroptosis and promotes lung tumorigenesis by affecting metabolic genes through DNA methylation and histone modification [87]. LSH activated the transcription of ferroptosis-related genes, including stearoyl-CoA desaturase 1 (SCD1) and fatty acid desaturases 2 (FADS2), by recruiting the WD repeat domain 76 (WDR76) to these genes' promoters. The expression of SCD1 and FADS2 affected the iron and lipid ROS accumulation, and SCD1 can also positively regulate CoQ10 production, thus suppresses lipid peroxide propagation.

The N-terminal of FSP1 contains a canonical myristoylation motif. By the mutation of the myristoylated modification site in FSP1, its antiferroptotic function was abolished; it was further demonstrated that myristoylation of FSP1 appears to be significant for its antiferroptotic function.

## 4. Ferroptosis and Drug Repurposing

The development of an intervention to effectively and selectively kill tumor cells is still defective, given the number of clinical trial failures in combination with the expensive costs for anticancer drugs, and there have been many reviews described preclinical drugs and compounds that can act as ferroptosis inducers. Our attention in this review was mainly focused on the existing drugs approved for other indications, which can be used for proferroptotic anticancer therapy. Here, we will review the mechanism and potential efficacy of the clinical drugs, which can be repurposed as ferroptosis inducer for cancer therapy.

### 4.1. Acetaminophen

Acetaminophen (APAP, paracetamol) is the most commonly used antipyretic and analgesic around the world [88]. Some non-small-cell lung cancer (NSCLC) cell lines are resistant to erastin-induced ferroptosis. Gai et al. reported that combined treatment of erastin and APAP inhibited NSCLC cell proliferation and promoted ferroptosis and apoptosis, accompanied with GSH attenuation and lipid peroxide accumulation [89]. Mechanistically, erastin and APAP sensitize tumor cells to ferroptosis by regulating nucleus translocation of NRF2 and the following downregulation of HMOX1 and transferrin, which led to elevated levels of LIP and thus making NSCLC cells sensitized to ferroptosis [23, 24, 89].

### 4.2. Artemisinin

Artemisinin and its derivatives (dihydroartemisinin, artesunate, artemether, and arteether), the active substances against malaria, have been explored as potential anticancer agents; their underlying mechanism for cancer therapy is still controversial [90]. Chen et al. reported that artemisinin compounds could sensitize tumor cells to ferroptosis [91]. Mechanistically, artemisinin compounds can induce lysosomal ferritin degradation in an autophagy-independent manner, following elevated levels of [Fe2^+^] LIP, thus sensitizing cells to ferroptosis. Furthermore, cellular labile iron concentration can be sensed by iron regulatory protein 1 (IRP1) and IRP2; these two proteins can dynamically regulate intracellular free iron levels by changing its affinity to mRNAs that contain iron-responsive element (IRE), thus regulating these kinds of gene expression.

When the iron level is at a normal level, IRP1/2 can bind to a mRNA class containing iron-responsive element (IRE), thus promoting these kinds of gene expression. For example, the expression of TFRC, which is having IRE, will be enhanced, whereas the expression of ferritin is suppressed. IRP-IRE signaling regulates iron homeostasis on a dynamic balance, and artemisinin compounds can impede IRP/IRE-controlled iron homeostasis to increase cellular free iron further. Accordingly, the mechanism of artemisinin compounds sensitizing tumor cells to ferroptosis is via regulating intracellular iron homeostasis. Previous clinical trials have shown the excellent safety and tolerability of artemisinin; therefore, using artemisinin as a proferroptotic agents can be a potential strategy for anticancer therapy [92].

### 4.3. Auranofin

Auranofin (AUR), an Au-containing compound, was first developed by Smith Kline and French Laboratories in 1976 [93] and was approved by the FDA to treat rheumatoid arthritis in 1985. Interestingly, recent researches reported that AUR could be a potential chemotherapeutic drug for several tumor types for its function as a strong thioredoxin reductase (TXNRD) inhibitor [94, 95]. Lee et al. reported that inhibition of TXNRD by AUR inhibited HCC cells' proliferation and induced apoptosis in vitro, which can also sensitize tumor cells to sorafenib [95].

AUR has been shown to act as a pan inhibitor of the TXNRD family, which serves as an essential ROS scavenger [96]. Recently, Yang et al. showed that high dose (25 mg/kg) of AUR could induce lipid peroxidation and ferroptosis through inhibition of thioredoxin reductase (TXNRD) activity [45]. Notably, in addition to AUR which acts as a ferroptosis inducer by TXNRD inhibition in high-dose situation, low-dose AUR can relieve iron overload by increasing hepcidin expression via the NF-*κ*B/IL-6/JAK-STAT pathway [45], which indicated that despite the new indication for proferroptotic anticancer therapy, AUR could also be repurposed to treat other hepcidin deficiency diseases. Ferrostatin-1 (Fer-1), a ferroptosis inhibitor, conferred significant protection against liver injury or other effects induced by high-dose AUR.

### 4.4. Cisplatin

Cisplatin is a chemotherapeutic agent, which has been a mainstay of cancer treatment for decades [97]. Notably, cisplatin is effective against various solid tumors, including ovarian, cervical, head-neck, NSCLC, and colorectal cancer. Unfortunately, many cancers initially respond to platinum treatment, but drug resistance often occurs when the tumor returns.

It was generally considered that cisplatin kills tumor cells by causing DNA damages via binding to DNA, therefore creating inter- or intrastrand crosslinks and finally leading to apoptosis. Recent studies have found that cisplatin acts as an inducer of apoptosis and ferroptosis in A549 and HCT116 cells [98]. Cisplatin promotes ferroptosis via GSH depletion and GPX4 inactivation; combination therapy of cisplatin and erastin showed a significant synergistic effect on their antitumor activity [98]. The resistance of tumor cells to cisplatin mainly affected cisplatin-induced apoptosis, but not cisplatin-induced ferroptosis. Therefore, targeting ferroptosis can be a promising way to alleviate tumor resistance to cisplatin.

### 4.5. Fenugreek

Fenugreek (trigonelline) is one of the oldest applied herbs. An emerging evidence indicates that Fenugreek can be used for antiatherosclerosis, anticancer, antidiabetes, and anti-inflammation. Recently, Roh et al. reported that trigonelline acts as an NRF2 inhibitor and sensitizes tumor cell to ferroptosis [99]. The inhibition of NRF2 by trigonelline repressed MT-1G expression, which improved the proferroptotic efficacy of sorafenib [100].

### 4.6. Haloperidol

Haloperidol, a sigma receptor 1 (S1R) antagonist, is a commonly used antipsychotic agent, which can be used for antiacute/chronic psychosis therapy. However, Bai et al. reported that haloperidol significantly promoted erastin- and sorafenib-induced cell death, which can be blocked by ferrostatin-1 but not ZVAD-FMK or necrosulfonamide [101]. S1R has been shown to function in antioxidant metabolism, and both erastin and sorafenib strongly enhanced S1P expression. Haloperidol treatment significantly increased the levels of intracellular free iron, facilitating GSH depletion and lipid peroxidation. Accordingly, haloperidol's combined treatment with sorafenib may be a novel strategy for hepatocellular carcinoma (HCC) therapy.

### 4.7. Neratinib

Neratinib is an HER-2 receptor tyrosine kinase inhibitor and is used in the extended adjuvant therapy of HER-2-positive early-stage breast cancer. Mechanistically, neratinib binds to the HER-2 receptor irreversibly, which thus blocked downstream signal transduction and cell cycle transition (arrest cell cycle at the G1-S phase) and finally repressed cell proliferation [102]. Nagpal et al. reported that neratinib promotes ferroptosis and inhibited brain metastasis in HER-2-positive breast cancer, but the underlying mechanism of how neratinib is inducing ferroptosis is obscure [103]. Neratinib-induced ferroptosis provides a new way for drug intervention. Further application of neratinib adjuvant therapy should be further assessed.

### 4.8. Siramesine Combined with Lapatinib

Siramesine, a sigma receptor 2 (S2R) ligand, was initially used for depression therapy [104] and was recently repurposed for cancer therapy [105]. Siramesine kills tumor cells via inducing cytoprotective autophagosome accumulation. Lapatinib is a small molecule inhibitor that can block several tyrosine kinase receptors' phosphorylation (EGFR, ErbB2, Erk1/2, and AKT kinases) and is generally used for advanced breast cancer therapy [106]. Ma et al. reported that the combination of siramesine and lapatinib synergistically induced ROS accumulation and ferroptosis [107], and this synergistic effect is mediated by lysosomes which release iron and proteasomes which degrade HO-1 [108]. These results suggest that altering iron homeostasis by clinical drugs could be a novel strategy for apoptosis-resistant cancer therapy.

### 4.9. Sorafenib

Sorafenib is an FDA-approved drug for the treatment of advanced hepatocellular carcinoma and primary kidney cancer. Sorafenib is a small molecular inhibitor that is uniquely targeting the Raf/Mek/Erk pathway and some other intracellular (CRAF, BRAF, and mutant BRAF) and cell surface kinases (KIT, FLT-3, VEGFR-2, VEGFR-3, and PDGFR-*β*) [109]. By inhibiting these kinases, cell proliferation and neoangiogenesis are inhibited.

Recent studies have identified that sorafenib could induce ferroptosis. The underlying mechanism of the correlation between ferroptosis and sorafenib is mainly in two ways. On the one hand, sorafenib inhibits the activity of system Xc^−^ thus causing the depletion of GSH and following lipid peroxidation [110]. On the other hand, the activation of NRF2 and MT-1G makes cells resistant to sorafenib. Under the treatment of sorafenib, the expression of p62 blocks NRF2 degradation and enhances the nuclear accumulation of NRF2, thus activating NADPH quinone's expression dehydrogenase1 (NQO1), FTH1, and HMOX1, which all can block the accumulation of ROS and lipid ROS in biomembranes. Genetic or pharmacologic inhibition of NRF2 expression in HCC cells increased the anticancer activity of sorafenib in vitro and tumor xenograft models [76]. Meanwhile, on the treatment of sorafenib, the expression of metallothionein- (MT-) 1G in HCC cells was significantly increased, which can hinder GSH attenuation-mediated lipid peroxidation and promote tumor cells resistant to sorafenib [100]. In summary, the above studies suggest that the combination of NRF2 or MT-1G inhibitor and sorafenib may prove to be a promising therapeutic strategy for cancer treatment.

### 4.10. Sulfasalazine

Sulfasalazine (SAS), the sulfonamide derivative, was firstly synthesized in 1940. It was clinically used to treat chronic inflammatory diseases and rheumatoid arthritis [111, 112]. Gout et al. first reported that SAS acts as a strong system Xc^−^ inhibitor and inhibits tumor cell proliferation by repressing GSH biosynthesis [113], and further study identified that SAS induces ferroptosis by inhibiting system Xc^−^ activity [1].

Accordingly, we summarized the existing clinically used drugs which can be repurposed for proferroptotic anticancer therapy (Table 2). The proferroptotic effects of these agents described above are all due to elevated oxidative stress, which is also the cause of their toxic effect. The comorbidity profile of individual patients may modulate response to therapy. Hence, when repurposing old drugs to target oxidative stress, various drug interactions should be considered. Inducing extensive ROS by these drugs can kill tumor cells efficiently, but may also be accompanied with serious toxic effect. How to apply them clinically needs further investigation.

In addition to the drug-drug interaction, drug-disease interactions also deserve special consideration. For example, overdose of APAP can result in organ toxicity, especially in the liver, and is the main cause of drug-induced liver injury and acute liver failure in many western countries [114]. The mechanism of APAP hepatoxicity is associated with APAP-induced lipid peroxidation and subsequent liver injury. Therefore, the application of APAP for proferroptotic anticancer therapy is limited in patients with liver dysfunction. Previous animal experiments and clinical trials show that fenugreek has a testicular toxicity and antifertility effects, which means that fenugreek is not recommended for pregnant women [115]. People with peanut allergy are also not recommended due to possible occurrence of chronic asthma [116].

## 5. Conclusions and Perspectives

Although substantial progress has been made about how tumor metabolism and oncogenic mutation regulate the sensitivity of ferroptosis, the extent to which the mutation profile affects the sensitivity to ferroptosis is still unclear. Given that tumor cells can persist and increase in such a high-oxidative stress environment, how can tumor cells evolve and adapt against increased oxidative stress? In addition to the anticancer context, why ferroptosis evolved the conserved and the fundamental role of ferroptosis in tumor development needs in-depth research. An emerging evidence has shown that ROS and lipid ROS play a different impact on cancer initiation, progression, and response to therapy; ferroptosis might act as a double-edged sword for cancer therapy. One hypothesis is that ferroptosis-sensitive states allow cancer cells to generate either prodeath or prosurvival lipid-derived mediators, which can act as a messenger to modulate intracellular and intercellular signaling pathways, so it is essential to understand the role of these lipid mediators before we can further develop a new strategy to kill tumor cells efficiently and selectively.

The epigenetic regulation of ferroptosis is poorly studied. What is the role of DNA methylation, RNA methylation, and posttranslational modification in ferroptosis regulation? How to use epigenome editing to manipulate tumor cells' sensitivity worth to explore? Likewise, developing a new strategy combined with immunotherapy and proferroptotic treatment may be an exciting way for drug-resistant cancer therapy.

## Figures and Tables

**Table 1:** p53-related ferroptotic genes.

Gene	Name	Function
ALOX12	Arachidonate 12-lipoxygenase, 12S type	A critical regulator of p53-dependent ferroptosis. Deletion of one AlOX12 allele is enough to abrogate p53-mediated ferroptosis [59].
CDKN2A	Cyclin-dependent kinase inhibitor 2A	CDKN2A activation sensitized tumor cells to ROS-induced ferroptosis [36].
DPP4	Dipeptidyl peptidase 4	DPP4 is critical for ferroptosis in p53-deficient colorectal cancer cells [72].
SAT1	Spermidine/spermine N1-acetyltransferase 1	p53-mediated activation of SAT1 sensitized tumor cells to ferroptosis in the presence of ROS stress. Knockdown of SAT1 partially rescued ROS-induced ferroptosis [66].
SOCS1	Suppressor of cytokine signaling 1	Expression of SOCS1 sensitized cells to ferroptosis inducer. This effect of SOCS1 was efficiently blocked by ferroptosis inhibitor. Expression of SOCS1 reduced the levels of GSH, explaining in part its ability to sensitize cells to ferroptosis [37].
SLC7A11	Solute carrier family 7 member 11	Elevated levels of SLC7A11 make cells resistant to erastin-induced ferroptosis. p53 negatively regulates SLC7A11 expression that sensitizes cells to undergo erastin-induced ferroptosis [117].
TP63	Tumor protein p63	Delta Np63 alpha can inhibit ferroptosis independent of p53. Overexpression protects cells from ferroptosis-inducing agents [118].
Mutated p53	Mutated p53 S47	The S47 mutation makes cell significantly resistant to agent-induced ferroptosis [119].
	Mutated p53 3KR	This p53 mutation type losses the ability to induce cell cycle arrest, senescence, and apoptosis, but still has the capability of SLC7A11 inhibition [64].
	p53 4KR98	p53 4KR98 is unable to induce ferroptosis, and its ability to block cancer proliferation is also abolished [117].
	p53 R273H and p53 R175H	These two mutations inhibit SLC7A11 expression by blocking NRF2-mediated SLC7A11 elevation [67–69].

**Table 2:** Old drugs used for proferroptotic anticancer therapy.

Old drugs	Target/function for ferroptosis
Acetaminophen	The combination of erastin and APAP can promote ferroptosis by NRF2 inhibition [89].
Artemisinin	Artemisinin compounds sensitize tumor cells to ferroptosis via impeding IRP/IRE-controlled iron homeostasis [91].
Auranofin	High dose of auranofin could induce lipid peroxidation and ferroptosis through TXNRD inhibition [45].
Cisplatin	Cisplatin promotes ferroptosis via GSH depletion and GPX4 inactivation [98].
Fenugreek	Fenugreek acts as an NRF2 inhibitor and sensitizes tumor cell to ferroptosis [99].
Haloperidol	Haloperidol treatment significantly increased the levels of intracellular free iron, facilitating GSH depletion and lipid peroxidation [101].
Neratinib	Neratinib promotes ferroptosis and inhibited brain metastasis in HER-2-positive breast cancer, but the underlying mechanism is still obscure [103].
Siramesine combined with lapatinib	The combination of siramesine and lapatinib synergistically induced ROS accumulation and ferroptosis, and this synergistic effect is mediated by lysosomes release iron and proteasomes degrade HO-1 [107, 108].
Sorafenib	Sorafenib induces ferroptosis via system Xc^−^ inhibition and following GSH depletion [100].
Sulfasalazine	Sulfasalazine acts as a strong system Xc^−^ inhibitor and induces ferroptosis by inhibiting GSH biosynthesis [1, 113].

## Data Availability

No data were used to support this study.
